# Spatial Variability of PAHs and Microbial Community Structure in Surrounding Surficial Soil of Coal-Fired Power Plants in Xuzhou, China

**DOI:** 10.3390/ijerph13090878

**Published:** 2016-09-02

**Authors:** Jing Ma, Wangyuan Zhang, Yi Chen, Shaoliang Zhang, Qiyan Feng, Huping Hou, Fu Chen

**Affiliations:** 1 Low Carbon Energy Institute, China University of Mining and Technology, Xuzhou 221008, Jiangsu, China; jingma2013@cumt.edu.cn (J.M.); slzhang@cumt.edu.cn (S.Z.); 2 School of Environment Science and Spatial Informatics, China University of Mining and Technology, Xuzhou 221008, Jiangsu, China; zwy2014zs@126.com (W.Z.); lucychenzqm@163.com (Y.C.); fqycumt@126.com (Q.F.); hphou@163.com (H.H.)

**Keywords:** polycyclic aromatic hydrocarbons, spatial distribution, source apportionment, high-throughput sequencing, soil microbial community

## Abstract

This work investigated the spatial profile and source analysis of polycyclic aromatic hydrocarbons (PAHs) in soil that surrounds coal-fired power plants in Xuzhou, China. High-throughput sequencing was employed to investigate the composition and structure of soil bacterial communities. The total concentration of 15 PAHs in the surface soils ranged from 164.87 to 3494.81 μg/kg dry weight. The spatial profile of PAHs was site-specific with a concentration of 1400.09–3494.81 μg/kg in Yaozhuang. Based on the qualitative and principal component analysis results, coal burning and vehicle emission were found to be the main sources of PAHs in the surface soils. The phylogenetic analysis revealed differences in bacterial community compositions among different sampling sites. *Proteobacteria* was the most abundant phylum, while *Acidobacteria* was the second most abundant. The orders of *Campylobacterales*, *Desulfobacterales* and *Hydrogenophilales* had the most significant differences in relative abundance among the sampling sites. The redundancy analysis revealed that the differences in bacterial communities could be explained by the organic matter content. They could also be explicated by the acenaphthene concentration with longer arrows. Furthermore, OTUs of *Proteobacteria* phylum plotted around particular samples were confirmed to have a different composition of *Proteobacteria* phylum among the sample sites. Evaluating the relationship between soil PAHs concentration and bacterial community composition may provide useful information for the remediation of PAH contaminated sites.

## 1. Introduction

As an important class of persistent organic pollutants (POPs), polycyclic aromatic hydrocarbons (PAHs) are mainly originated from incomplete combustion of fossil fuels, forest fires, crop straw and other organic materials that continuously burn [1,2]. The derivatives from the diagenesis of crude oil and organic matter under anoxic conditions are also the main PAH sources [3]. PAHs are ubiquitous environmental contaminants which are widely distributed in various environments, such as the atmosphere, water, soils and sediments [4,5]. As a rapidly developing country, China has the highest PAH emissions in the world. PAs emission in China was around 114 Gg in 2004, constituting 22% of total global emissions [6]. 

Due to the carcinogenic properties and persistence of PAHs in the environment, many countries have taken efforts in reducing PAH emissions. Understanding the contribution of PAH emission sources is important for proper management of PAH levels in the environment. The study of PAH distribution in representative locations can assist in understanding the relation between PAH characteristics and their anthropogenic influence. Natural (such as oil seeps and forest fires) and anthropogenic (such as fossil fuels and combustion) inputs can be traced when PAHs are used alongside with related hydrocarbon marker compounds [7]. The source discrimination of PAHs is facilitated by many approaches, such as PAH ratio methods [8], multivariate analysis [9] and isotope ratio mass spectrometry [10]. Diagnostic ratios can be used for qualitative analysis of various PAH component sources and can primarily give valuable information primarily when there are one or two main sources. However, in the vast majority of cases, more details about the source apportionment are essential for controlling PAH pollution. It has been reported that it is difficult for finer differentiation of possible combustion sources due to the overlap. It has been mainly confined to the laboratory or urban locations [11]. Principal component analysis (PCA) provides a recognition capability, which could improve the source discrimination. PCA is a commonly used tool for multi-variable analysis. However, it is not restricted to the study of receptor model in the research of environmental source apportionment [12]. 

Once introduced into the soil, PAHs can be decomposed via biotic or abiotic processes, or it can be transferred to soil profiles and groundwater through runoff and leaching [3]. As the most abundant and diverse groups of soil microorganisms, bacteria play an indispensable role in soil ecology, particularly due to their central role in decomposition. Due to their enormous metabolic versatility, they also serve as keystones in biogeochemical cycling [13]. Microbial degradation is a primary process occurring in the natural PAH depletion processes. Bacteria that have excellent PAH-degrading ability are widespread in various environmental media. According to several reports, indigenous microbial communities are important for the success of bioremediation. However, little is known about the difference of microbial composition and structures among the real, historically contaminated soil [14]. Adequate information of microbial consortia structure is useful for monitoring the occurrence of real time biodegradation processes, as well as for carrying out accurate risk assessment in situ. Studies on bacterial community structure associated with bioremediation of PAH-contaminated soils have been reported using traditional standard biochemical tests [15]. Most soil microorganisms cannot be cultivated at present by laboratory approaches [16]. Therefore, several molecular biological techniques such as terminal restriction fragment length polymorphism (T-RFLP) and polymerase chain reaction denaturing gradient gel electrophoresis (PCR-DGGE) have been employed to characterize bacterial community structures [17,18]. However, unlike the DGGE method, the T-RFLP technique could not be used for map hybridization or direct cloning and sequencing analysis. The single enzyme digestion fragment is not accurate enough to identify the genus level in the database. In addition, the experimental procedures for DGGE are cumbersome and time consuming. Some analyzing fragments are easily lost in the repeated PCR process of DGGE. Also, these expensive and time-costly techniques can only provide limited information on community structures. In recent years, high-throughput sequencing techniques, such as Illumina sequencing, have enabled more comprehensive detection of microbial community changes and minor populations [19,20]. This approach has been used to decipher microbial communities in distinct samples, such as samples taken from polychlorinated biphenyl contaminated environments [21], river water [22] and iron mining soil [23]. 

Xuzhou is the most important coal production base and an important thermal power plant base in east China. It has more than 80 years of history in producing and using coal. It is generally believed that coal combustion in coal-fired power plants is the main source of PAH pollution in surrounding farmland. However, with the rapid development of automobiles in China, vast petroleum consumption overlaps with human-intensive activity areas. Therefore, it is crucial to the repair strategy formulation to determine whether or not coal combustion is the only pollution source for coal-fired power plants in the surrounding farmland. Based on these reasons, this work investigated the surrounding soil of coal-fired power plants to confirm the source and to provide a scientific basis for soil remediation. Moreover, illumina sequencing was applied to examine microbial community profiles in different sampling sites. This study is intended to provide broad insights for assessing the impact of coal-fired power plants on PAHs emission and surrounding environments.

## 2. Materials and Methods

### 2.1. Site Description and Sampling

**Figure 1:**
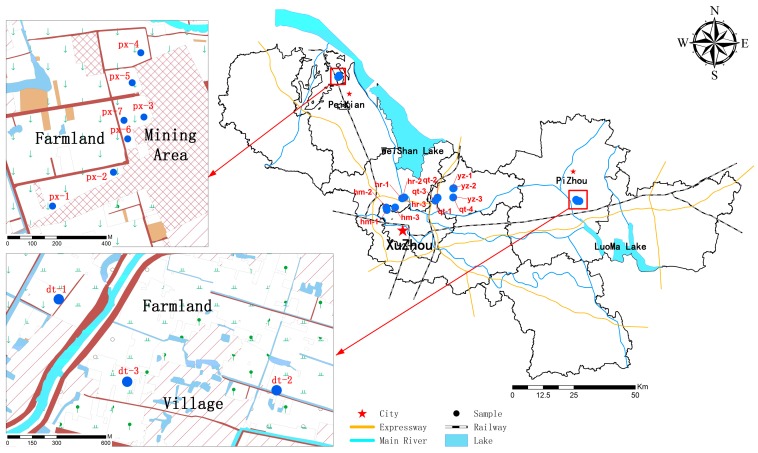
Map showing the sampling locations in Xuzhou city.

Figure 1 demonstrates the locations of sampling sites. These location sites include the following: there were seven samples (px1–px7) collected from the Datun electric power plant, three samples (hr1–hr3) near the Huarun power plant, three samples (dt1–dt3) along the Datang power plant, three samples (hm1–hm3) along the Huamei plant, three samples (yz1–yz3) near the Yaozhuang power plant and four samples (qt1–qt4) near the Quantai power plant. 

Soil samples were collected in November 2014. The top 10 cm layers were carefully removed with an iron shovel. All samples were packed into aluminum boxes and immediately stored at 4 °C just for 1–3 days before being extracted. After air-drying at room temperature, the soils were sieved through a 100-mesh sieve. Total organic matter (TOM) was determined through the Walkley-Black wet oxidation method [24]. 

### 2.2. Chemicals

A mixture of 16 USEPA priority PAHs standards solution [naphthalene (Nap), acenaphthylene (Acy), acenaphthene (Ace), fluorene (Fle), phenanthrene (Phe), anthracene(Anth), fluoranthene (Fla), pyrene (Pyr), benzo[a]anthracene (BaA), chrysene (Chr), benzo[b]fluoranthene (BbF), benzo[k]fluoranthene (BkF), benzo[a]pyrene (BaP), indeno[1,2,3-c,d]pyrene (Ind), dibenz [a,h]anthracene (DBA), and benzo[g,h,i]perylene (BgP)] was purchased from TCI Development Co., Ltd. (Beijing, China). All organic solvents are HPLC grade and purchased from SK Chemicals (Ulsan, Korea). In order to exclude impurities before use, analytical-grade anhydrous Na_2_SO_4_ and silica gel were activated at 320 °C to exclude impurities before use. 

### 2.3. PAH Analysis

One gram of sieved soil was combined with a 4 mL mixture of dichloromethane and acetone (*v*:*v*, 1:1) and then sonicated for 30 min. The process was repeated three times by removing the extract and adding fresh solvent. All extracts were combined and concentrated by rotary evaporation. Then, eluting through a chromatography column, which was filled with silica gel and anhydrous Na_2_SO_4_, purified them. The elution liquid was dried under nitrogen, and the residue was dissolved in 1 mL acetonitrile for HPLC analysis [2].

The PAHs were analyzed by HPLC (Shimadzu-UFLC-10A, Shimadzu (China) Co., Shanghai, China) with a fluorescence detector. The PAH special column (Waters, 250 mm × 4.5 mm, 5 μm) was packed into the analyzer. The injection volume was only 1 μL. Also, the column temperature was 35 °C. The gradient operation procedure was conducted as follows: At the beginning, 55% acetonitrile was used, 10 min later, 70% acetonitrile was used for a linear gradient at100% acetonitrile for 15 min. This was kept for 5 min at 100% acetonitrile. A linear gradient (2 min) back to starting conditions was followed by a 3 min run to stop. In this study, a fluorescence detector quantified 15 PAHs, excluding acenaphthylene. 

In this study, the Phe/Anth, Fla/Pyr, Fla/(Fla + Pyr) and Ind/(Ind + BgP) values were used to determine the PAH sources.

### 2.4. Microbial Community Analyses

According to the instruction manual of the FastDNA^TM^ SPIN kit, DNA was extracted from 0.5 g soils (MP Biomedicals, Solon, OH, USA) in duplicate which have been stored at 4 °C. Using an Eppendorf thermal cycler (Model 5332), the V4 region of bacterial 16S rRNA genes were amplified. The temperature program consisted of initial denaturing at 98 °C for 5 min. This was followed by 25 cycles at 98 °C for 30 s, 50 °C for 30 s, 72 °C for 30 s. Then there was a final extension step at 72 °C for 5 min. The primer set for the amplification was 520F (5’-barcode + GCACCTAAYTGGGYDTAAAGNG-3’) and 802R (5’-TACNVGGGTATCTAATCC-3’). The barcode was a seven-base sequence unique to each sample.

The PCR reaction was conducted in triplicate in a 25-μL mixture containing 5 μL of 5 × Q5 Reaction Buffer, 5 μL of 5 × Q5 GC high Enhancer, 2 μL of 2.5 mMdNTPs, 1 μL of each primer (10 μM), 0.25 μL of Q5 Polymerase, 8.75 μL of sterilizing ultrapure water and 2 μL of template DNA (20 ng/μL). Three PCR products per sample were pooled and purified with the AxyPrep DNA Gel Extraction Kit (Axygen Biosciences, Union City, CA, USA) in accordance with the instruction manual. Quantification of purified PCR product was performed with a Quant-iTPico Green dsDNA Assay Kit (Invitrogen, Carlsbad, CA, USA). The purified amplicons were pooled in equimolar and paired-end sequenced (2 × 300 bp) on an IlluminaMiSeq platform (Personalbio, Shanghai, China) according to standard protocols. Raw FASTQ files were de-multiplexed and quality-filtered using QIIME (version 1.7.0) with the following criteria: (1) 300-bp reads were truncated at any site that acquired an average quality score of >20 over a 10-bp sliding window. Truncated reads shorter than 150 bp were discarded; (2) reads that contain ambiguous characters and two nucleotides that mismatched in primer matching were removed; (3) according to overlapped sequences, overlapping sequences that are longer than 10 bp would be assembled. Unassembled reads were discarded. Operational taxonomic units (OTUs) having 97% similarity were clustered using the Uparse soft (version 7.1). The Uchime method was used to identify and remove the chimeric sequences [23]. Greengene was used as the annotation database [25]. Sequences that could not be allocated into any known groups were defined as others.

### 2.5. Data Analyses

PCA was employed to analyze the data groups of PAHs concentrations in order to compare the profiles of each potential PAH source using SPSS 18.0 software (Cabit Information Technology Co., Shanghai, China). Redundancy analysis (RDA) with the Monte Carlo permutation’s test (499 permutations) was used to evaluate the correlation between soil environmental factors and bacterial community structures through the use of the software Canoco (Cabit Information Technology Co., Shanghai, China) for Windows 4.5.

## 3. Results and Discussion

### 3.1. Spatial Distribution of PAHs in the Surface Soil

The contamination level of PAHs in soil can be divided into four classes: no contamination (total PAHs concentration < 200 μg/kg), slight contamination (200 < total PAHs concentration < 600 μg/kg), moderate contamination (600 < total PAHs concentration < 1000 μg/kg) and severe contamination (total PAHs concentration > 1000 μg/kg) [26]. The condition of surface soils can reflect the current soil contamination status. 

**Figure 2:**
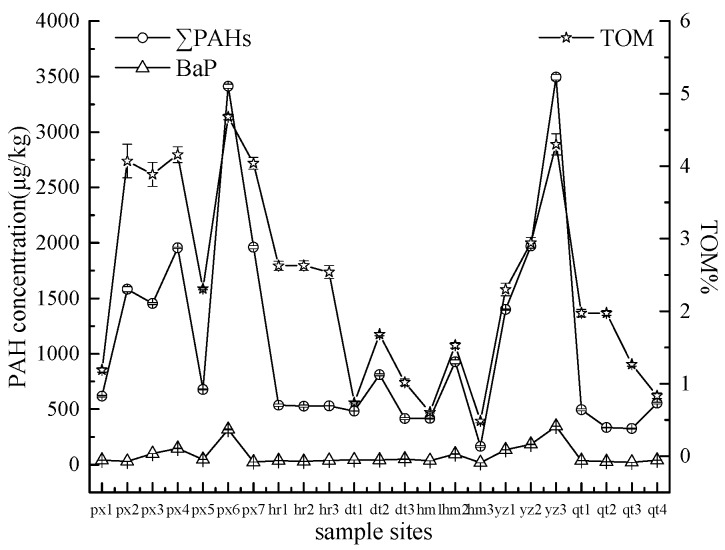
PAHs concentrations and TOM content in each soil sample (see supplementary material).

As shown in Figure 2, the total concentration of 15 PAHs in soils ranged from 164.87 to 3494.81 μg/kg dry weight with an average concentration of 1089.69 μg/kg. According to the grading criteria, the PAHs pollution levels in the study area rangedfrom no pollution to severe pollution. At sites px6 and yz3, two highest PAHs concentration were observed (3412.64 and 3494.81 μg/kg). Compared with the PAH concentrations reported in farmland or suburb soil of Korea (236 μg/kg), Switzerland (225 μg/kg), Malaysia (155 μg/kg), New Orleans (242 μg/kg) and Shanghai (665.8 μg/kg) [27,28,29,30,31], the average concentration of 1089.69 μg/kg found in this study was much higher. However, compared with the PAH concentration of 1840 μg/kg in urban surface soil of Tianjin [32], the PAH concentration in this study was lower. According to the soil quality standard of Canada, the reference value of BaP in agricultural land is 100 μg/kg [33]. BaP has also been classified as group 1 toxic in regard toits carcinogenicity to humans by the IARC (International Agency for Research on Cancer) [34]. In this work, BaP concentrations in five samples (px4, px6, yz1, yz2 and yz3) exceeded the reference value (Figure 2). 

TOM is an important factor that can impact the transport and fate of PAHs in soils [11]. The relatively low concentrations of PAHs at power plants hr, dt, hm and qt showed consistentlow levels of TOM (Figure 2). A linear regression analysis indicated that the total concentrations of PAHs were significantly correlated with the TOM contents (*r* = 0.894, *p* < 0.01). However, the relatively low concentration of PAHs at site px5 with higher TOM content suggested that there existed other factors, such as the application of livestock manure, which may affect TOM levels.

### 3.2. Source Analysis via PAHs Constitution

**Figure 3:**
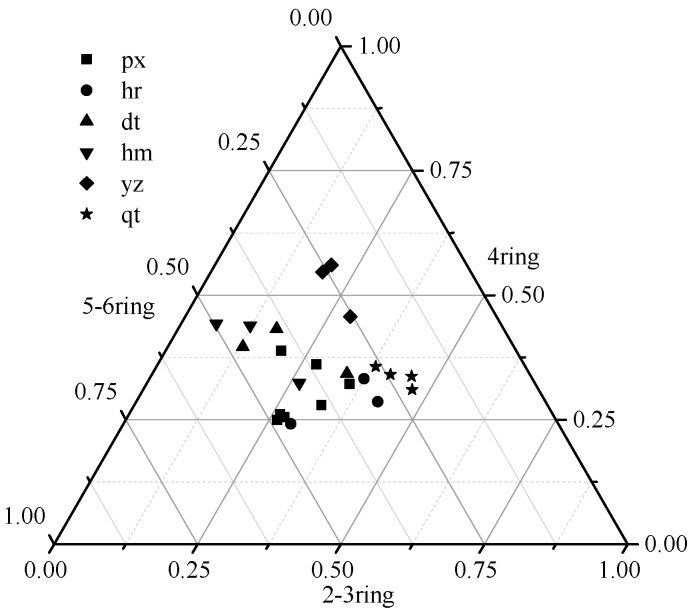
Triangle graph of percentage density for 15 PAHs in the sampling sites.

According to ring numbers, 15 PAHs were classified into three classes: 2–3 ring, 4-ring, and 5–6 ring compositions. These three classes represented low, medium and high molecular weight PAHs, respectively. Figure 3 shows the composite model of 15 PAHs. 4-ring PAHs comprised more than 50% of the total PAHs in all sampling sites, in particularly sites dt, hm and yz, which all had two samples with more than 75% 4-ring composition. For the composition of 2–3 ring and 5–6 ring PAHs, significant discrepancies were detected between sampling sites. Sites px and hm had higher 5–6 ring PAHs composition, while sites hr and qt had higher 2–3 ring composition. These findings indicated that there may be different sources for the PAHs in the soils surrounding different sampling sites. The component percentages of PAHs at sites hr and qt were similar, suggesting their similar pollution sources. Relative to low molecular weight PAHs (2–3 rings), PAHs with higher molecular weights (≥4 rings) adsorb more easily on particulate matter, such as soil particles [35]. 

### 3.3. Qualitative Research of PAHs Sources

The analysis and confirmation of PAHs sources are important to regulate and manage their input, as well as their distribution liability for remediation activity. Based on the characteristics of PAHs compositions and allocation patterns, the sources of anthropogenic PAHs that were formed primarily through emission and combustion of incomplete combusted materials, can be differentiated by individual PAH ratios [8].

**Figure 4:**
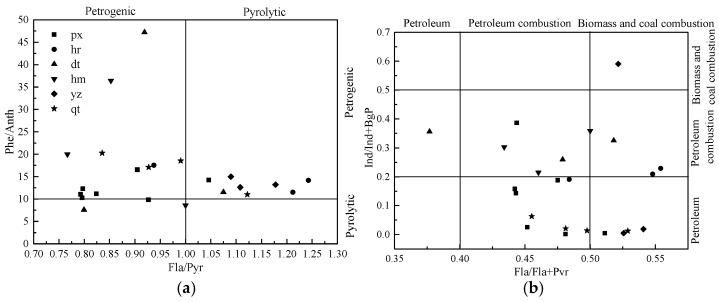
Source analysis of PAHs through diagnostic ratios. (**a**) Plot of Fla/Pry vs. Phe/Anth; (**b**) Plot of Fla/(Fla + Pyr) vs. Ind/(Ind + BgP).

As isomers, Anth is thermodynamically more unstable than Phe. The Phe/Anth value >10 suggests petrogenic contamination, whereas the value <10 implies a pyrolytic source [11]. Pyr is thermodynamically more stable than Fla. Fla/Pyr > 1 is a feature of the pyrolytic process, while Fla/Pyr < 1 indicates a petroleum origin [7]. As shown in Figure 4a, the Phe/Anth ratios of soil samples ranged from 7.58 to 47.23. All the samples had Phe/Anth ratios higher than 10, except for sites px, dt and hm. This suggested that soil PAHs might be mainly from petrogenic sources. Fla/Pyr values in sites px, dt, hm and qt were mostly less than 1, indicating petrogenic sources of PAHs. This was in agreement with the results of Phe/Anth ratios (Figure 4a). However, the range of Fla/Pyr ratios in samples of sites hr and yz was from 0.94 to 1.24, suggesting a mix of petrogenic and pyrogenic sources. 

For Fla/(Fla + Pyr), a ratio <0.40 suggests petrogenic origins, a ratio >0.50 implies coal, grass, and wood combustion origins, and a ratio of 0.40–0.45 has been described for gasoline engine exhausts [8]. Different ratios of Ind/(Ind + BgP) have been connected to diesel and gasoline vehicles [36]. A ratio below 0.20 is ascribed to petrogenic origins. A ratio above 0.50 indicates wood and coal combustion, whereas the ratio between 0.20 and 0.50 is a trait of petroleum burning performance. As shown in Figure 4b, the ratios of Fla/(Fla + Pyr) in all sampling sites were higher than 0.40, except for one sample from site dt, with an average value of 0.49. This suggested that these sites had the mixed pattern of petroleum and biomass burning sources. Several samples from site px had the ratio of Fla/(Fla + Pyr) between 0.40 and 0.45, which implied there was a gasoline fuel combustion source. Also as shown in Figure 4b, the ratio of Ind/(Ind + BgP) was mostly lower than 0.50, particularly, the ratio for sites qt and px were lower than 0.20, indicating petrogenic origins. The Ind/(Ind + BgP) ratio of samples from site hr, dt and hm was mostly between 0.20 and 0.50, suggesting petroleum burning sources. According to PAHs source patterns revealed by the specific values of Phe/Anth, Fla/Pyr, Fla/(Fla + Pyr) and Ind/(Ind + BgP), PAHs in the sampling sites were primarily from the mixed sources of petroleum and petroleum combustion. 

### 3.4. Source Estimates by PCA

The objective of PCA is to perform the diversification of PAHs concentrations among sampling sites with the minimum number of factors. The weighted linear combination of the original variablesis the first factor accounting for the greatest variability. Each subsequent factor is responsible for less variability than the former one. 

The results of PCA based on PAHs concentrations are listed in Table 1. Three principal components (PCs) were extracted for soils, which accounted for more than 97% of the variance in the data set. The cumulative variances occupied 88.236% of the total variation. The three factors were 58.132%, 22.334%, and 7.770%, respectively. Each variation’s load value > 0.7 was highlighted in red bold face.

Factor 1 is heavily weighted by Fle, Phe, Anth, Fla, Pyr, BaA, Chr, BbF and BaP. Previous studies reported the following as dominant, coal combustion profiles: Fla, Pyr, Phe, Anth and BaA [11,37]. Thus, factor 1 seemed to be associated with coal burning.

Factor 2 is predominately weighted by Nap and Ind. Nap is the lightest PAHs quantified, which could be related to the unburned fossil fuels and other sources [38]. Ind has been detected from both diesel and gas vehicle exhaust [39]. BgP, the third highest loading value of factor 2, has been confirmed as indicators of automobile emission [40]. Therefore, factor 2 mainly represents vehicle emissions. 

Factor 3 shows a good relevance to DBA, which has been reported to be related to automotive engine exhaust [41]. 

Generally, the PCA results were in accordance with the results depicted by diagnostic ratios. The PAHs in most soil samples were from both coal combustion and vehicle exhaust. Also, PCA results indicated that PAHs distribution in the soil samples could be related to the surrounding coal-fired plants. 

### 3.5. Bacterial Taxonomy Composition

**Figure 5:**
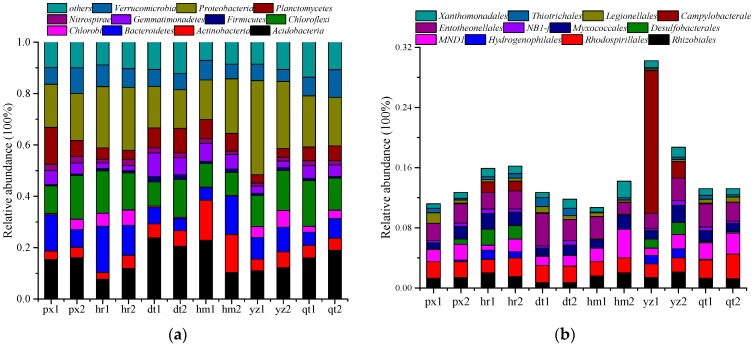
Taxonomic distributions of 12 soil samples. (**a**) Phylum profile; (**b**) Order distribution of phylum *Proteobacteria*.

**Figure 6:**
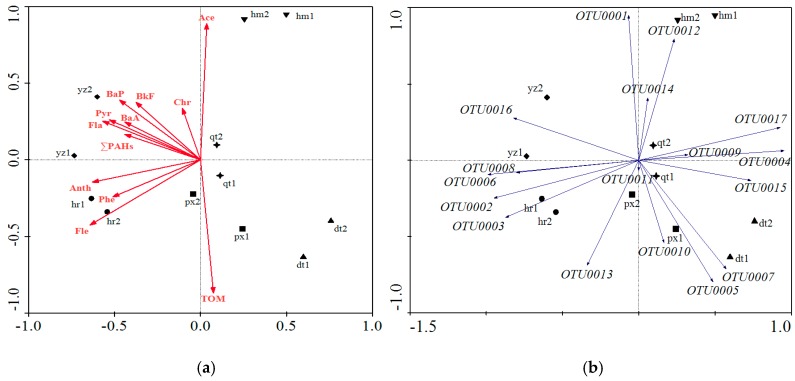
Redundancy analysis showed the correlation between bacterial consortia and soil environment factors (**a**) and some frequent OTUs of phylum Proteobacteria (**b**).

The bacterial OTUs obtained from Illumina sequencing of 16S rRNA genes were classified into 35 different phyla or 254 orders. Figure 5a showed that *Acidobacteria*, *Actinobacteria*, *Bacteroidetes*, *Chloroflexi*, *Gemmatimonadetes*, *Planctomycetes*, *Proteobacteria* and *Verrucomicrobia* phyla accounted for more than 80% of the total reads in every sample. Phyla that occupied less than 1% of the bacterial communities were not shown. *Proteobacteria* was the most abundant phylum (Figure 5a), ranging from 15.1% to 36.6% in the samples. *Acidobacteria*, the second most abundant phylum, occupied about 15.6% in all samples. In general, bacterial community structures varied among different sampling sites (Figure 5a). The trend was more evident as revealed by RDA analysis with samples that came from around the same power plants grouped nearer to each other (Figure 6). 

The orders of *Campylobacterales, Desulfobacterales* and *Hydrogenophilales* from phylum *Proteobacteria* showed the most obvious distinction of relative abundances among the sampling sites (Figure 5b). Relative abundances of the three orders were higher in sites hr and yz than others. It has been reported that *Desulfobacterales* are sulfate reducing bacteria and strictly anaerobic [42]. In contrast, the relative abundances of *Rhodospirillale s*and *Entotheonellales* were similar across all the samples. Bacterial consortia enriched from natural communitieshad been reported with reduced complexity. This facilitated the relationship between specific populations and functions. Sun et al. [43] identified *Alphaproteobacteria* (*Rhizobium*) and *Betaproteobacteria* (*Hydrogenophaga*) as the most abundant bacterial components of a pyrene-degrading consortium. Jones et al. [44] also identified that *Cupriavidus* (*Betaproteobacteria*) and *Luteimonas* (*Gammaproteobacteria*) had a close correlation with benzo(a)pyrene cometabolism in the PAHs polluted soil. Getting information about the response of community members to the stimulation can guide the interpretation and development of remediation approaches. Handley et al. [45] employed a high-density microarray (PhyloChip) to comprehensively determine community membership and abundance patterns among a suite of samples associated with uranium bioremediation experiments. Results showed that *Campylobacteriales* thrived as a response to acetate amendments and they were associated with Fe (III) and sulfate reduction. Zhao et al. [46] have detected *Campylobacteriales* in reactors with multiple electron acceptors, under sulfur cycling conditions. It was also reported that *Campylobacteriales* were detected in wastewater, microbial fuel cells (MFCs) and microbial electrolysis cells (MECs) studies [47]. All these studies imply that *Campylobacteriales* have a significant role in decomposing organic matters. Higher relative abundances of *Campylobacterales*, *Desulfobacterales* and *Hydrogenophilales* in sites hr and yzmight be related to higher PAH concentration. The results also suggested that we might get high efficiency degrading strains from yz sites. 

This study confirms the important role of *Proteobacteria* in PAHs polluted soils. In this study, the high-throughput 16S RNA gene sequencing provides detailed information on the taxa comprising of bacterial community. However, it gave little insight into the functional role of the *Proteobacteria* phylum, especially *Campylobacterales*, *Desulfobacterales* and *Hydrogenophilales*. Future research will be of interest in pursuing a deeper understanding of the metabolism and diversity of this phylum.

### 3.6. Correlation between Bacterial Community Structure and PAH Content and TOM

The redundancy analysis (RDA) ordination diagram is plotted in Figure 6a. The goodness of fit statistics for environmental variables showed that the ordination was highly correlated with TOM, Ace, Fle and other PAHs (*p* < 0.05) with long arrows (Figure 6a). Among the environmental factors, TOM, Ace, Chr and Fle were highly correlated with Axis 1, which explained most of the variations in the bacterial community. This is not surprising because TOM was shown to be correlated with PAH concentration, as revealed in Figure 2. The arrows of TOM and Ace were longer, indicating that they were the most important variables regarding bacterial community ordination. The TOM content was highly correlated with bacterial community structures in site px (Figure 6a). Fle, Phe and Anth distinguished the bacterial community of site hr from other sites (Figure 6a). Moreover, bacterial communities of sites hr and yz showed more distinctions compared with other sites with different PAHs contents as revealed in Figure 6a. Other environmental factors such as pH and EC values have been measured too. However, no regularity has been found between pH, EC values and variation of bacterial phyla (data not shown). RDA ordination showed several OTUs of *Proteobacteria* particularly characterized some communities according to the bacterial community composition (Figure 6b). Sites px, qt and dt were associated with OTUs belonging to *Deltaproteobacteria* and *Gammaproteobacteria* classes: *Entotheonellaceae* (OTU0004), *Syntrophobacteraceae* (OTU0017), *Piscirickettsiaceae* (OTU0005 and OTU0007) and *Sinobacteraceae* family (OTU0015). Site hm was characterized by OTUs related to *Alphaproteobacteria* (OTU0001) and *Betaproteobacteria* classes (OTU00012). Sites hr and yz were related to *Deltaproteobacteria* (OTU0002 and OTU0003), *Betaproteobacteria* (OTU0006) and *Alphaproteobacteria* (OTU0016). The arrows of OTU0001, OTU0002, OTU0003, OTU0005, OTU0006 and OTU0017 were longer, which indicated that these six OTUs were the most important variable OTUs characterizing the sampling sites. The relationship between OTUs and samples showed that the abundance order of OTU0001 in each site was hm > yz > qt > hr ≈ px > dt. The abundance order of OTU0002, OTU0003 and OTU0006 was hr ≈ yz > qt ≈ px > hm ≈ dt. The abundance order of OTU0017 in each site was hm ≈ dt > qt ≈ px > hr ≈ yz. These results indicated that the *Proteobacteria* phylum had significantly different compositions among the sampling sites. Moreover, some researches revealed that *Betaproteobacteria* (*Acidovorax*, and *Hydrogenophaga*) might have a crucial role in the early response to PAH exposure 16. Moreover, other authors also revealed groups of phenanthrene, anthracene and pyrene-degrading *Proteobacteria* [48,49,50], whose abundance and response to simulated bioremediation was demonstrated by barcoded pyrosequencing [14]. 

## 4. Conclusions

This work provides important information on the distribution tendency of PAHs and the soil microbial community in soil samples surrounding a few coal-fired power plants. A positive association was found between PAHs and soil organic matter. The most affluent PAH congener of all samples was considered to be the four-ring PAHs. Most PAHs were primarily from coal burning or vehicle exhaust, which may be related to the surrounding coal-fired plants. Shifts in diversity and composition of bacterial community were also defined. Soil organic matter was correlated with PAH concentrations. The correlation between soil organic matter and PAHs concentrations may account for the differences among bacterial communities. *Proteobacteria* phylum was the most abundant group and had the greatest variation. The obvious changing trend related to *Proteobacteria* may provide useful information for further research on the relationship between PAHs and bacterial communities. This phylum can be also considered an important biological resource for bioremediation of PAH-contaminated soil.

## Figures and Tables

**Table 1:** Rotated composition loading values of three principal components (PCs) for PAH compositional analysis.

Variable	PC1	PC2	PC3
Nap	0.423	**0.727**	0.381
Ace	0.573	0.165	−0.032
Fle	**0.816**	0.348	−0.391
Phe	**0.932**	0.153	−0.243
Anth	**0.933**	0.212	−0.149
Fla	**0.852**	−0.518	−0.042
Pyr	**0.935**	−0.343	−0.037
BaA	**0.925**	−0.367	−0.012
Chr	**0.741**	−0.560	0.269
BbF	**0.942**	0.288	−0.046
BkF	0.572	−0.762	0.281
BaP	**0.894**	−0.263	−0.022
Ind	0.623	**0.734**	−0.152
DBA	0.443	0.345	**0.754**
BgP	0.452	0.616	0.192
Explained variance (%)	58.132	22.334	7.770

Note: PCA loading values higher than 0.7 are in bold.

## Supplementary Materials

The following are available online at www.mdpi.com/1660-4601/13/9/878/s1.
